# Rationale and protocol for an observational study of *in vivo* stress experiences and real-time cardiovascular responses among young, black women: The DYNAMIC study

**DOI:** 10.1371/journal.pone.0330508

**Published:** 2025-09-29

**Authors:** Anika L. Hines, Fota Sall, Shawn Utsey, Robert Perera, Ron K. Evans, Lisa A. Cooper, Jessica Gokee LaRose

**Affiliations:** 1 Department of Health Policy, Virginia Commonwealth University, School of Public Health, Richmond, Virginia, United States of America; 2 Division of General Internal Medicine, Johns Hopkins University, School of Medicine, Baltimore, Maryland, United States of America; 3 Department of Psychology, Virginia Commonwealth University, College of Humanities and Sciences, Richmond, Virginia, United States of America; 4 Department of Biostatistics, Virginia Commonwealth University, School of Public Health, Richmond, Virginia, United States of America; 5 College of Humanities and Sciences, Department of Kinesiology and Health Sciences, Virginia Commonwealth University, Richmond, Virginia, United States of America; 6 Bloomberg School of Public Health, Johns Hopkins University, Baltimore, Maryland, United States of America; 7 Department of Social and Behavioral Sciences, Virginia Commonwealth University, School of Public Health, Richmond, Virginia, United States of America; Public Library of Science, UNITED KINGDOM OF GREAT BRITAIN AND NORTHERN IRELAND

## Abstract

**Background:**

Chronic stress is thought to contribute to racial disparities in cardiovascular morbidity and mortality among women. Intervention development has been stalled by complex issues regarding stress measurement as well as the correlation of varying stress experiences with proximal cardiac responses in naturalistic environments.

**Methods:**

The Designing Young Adult Interventions to Address and Mitigate Inequities in Cardiovascular Health (DYNAMIC) Study is an observational study of the stress experiences of young Black women reported *in vivo* and within naturalistic context. The study will enroll up to 50 participants aged 18 to 39 years old. Participants will undergo a 14-day protocol, including completion of random surveys regarding stressor administered via ecological momentary assessment (EMA) while wearing a 24-hour ambulatory ECG monitoring patch and actigraph watch to capture a continuous feed of physiological responses. Participants will also measure and log wakening and bedtime blood pressure readings using a validated cuff.

**Discussion:**

The study addresses ongoing challenges to validity in correlating stress to cardiovascular outcomes. The combination of EMA surveys and continuous physiological monitoring provides rich data regarding stress response, including frequency, duration, and attribution to specific stress stimuli. The primary outcomes of the study are heart rate variability and blood pressure. The secondary outcome of this study is ideal cardiovascular health—an index of measures predicting healthy cardiovascular aging.

**Conclusion:**

Findings from this study may be used to inform responsive, tailored interventions for young Black women towards the aims of primordial prevention and early intervention to promote cardiovascular health and reduce disparities.

## Introduction

Cardiovascular disease (CVD) is the leading cause of death among women [1]. Black women have higher risk of developing CVD and at earlier ages compared to white women [2,3] as well as poorer cardiovascular health as measured by key health behaviors and risk factors, indicative of healthy cardiovascular aging [4–6]. Experiencing disproportionately higher levels of chronic stress—as measured by biomarkers of inflammation and dysregulation—is thought to be a root cause of these disparities though mechanisms are not fully explicated [7–9].

Structural stressors have direct effects on health and well-being via systemic limitations of access to resources and opportunities [10–13] as well as indirect effects through increasing vulnerability to individual stressors [13]. Neighborhood environment and racial discrimination have been shown to partially explain differences in cardiovascular health among Black and white women [5]. Stress coping strategies may also have detrimental effects on physiological outcomes even when they provide psychological reprieve. For example, anticipatory vigilant coping—the practice of preparing or “bracing” oneself for potential mistreatment—may exacerbate hypertension risk [14,15]. The Superwoman Schema—emphasizing strength, resistance, determination, helping others, and suppression of emotion, characterized among Black women [16]—may serve as either an asset or a liability in terms of psychological stress and physiological outcomes [17–20].

Despite mounting evidence that psychosocial stress is a contender in improving cardiovascular outcomes among Black women, few tailored interventions exist [21,22]. To effectively prevent cardiovascular disease, interrupt its development, and/or thwart CVD exacerbation, better understanding of how stressors unfold in daily life and naturalistic environments is required, including multiple daily measurements of self-reported stress and continuously assessed cardiovascular measures [23]. Measurement of stress is complex, especially for Black women who may underreport stress and whose lived experiences may not be represented in mainstream instruments [21]. In addition, the most frequently used instruments are employed to assess stress over the past month, which complicates correlation to proximal physiological cardiovascular responses [24].

The objective of this study is to understand the associations among stressors and cardiovascular responses among young, Black women in real-time and context over a 14-day period using rigorous empirical methods. Evidence may be used to identify and refine targets for intervention within this vulnerable population of potentially high public health and cardiovascular health impact.

## Methods

### Overview of study design

The Designing Young Adult Interventions to Address and Mitigate Inequities in Cardiovascular Health (DYNAMIC) Study is an observational study of the stress experiences of young Black women reported *in vivo* and within naturalistic context. DYNAMIC will recruit up to 50 self-identified Black adult women under the age of 40 years old. Participants will undergo a 14-day protocol, including random surveys regarding experienced stressors administered via ecological momentary assessment (EMA) while wearing a 24-hour ambulatory ECG monitoring patch and actigraph watch to capture a continuous feed of physiological responses. Participants will also record their wakening blood pressure (BP) and sleep quality through a baseline and close-out survey administered daily via the EMA application. The primary outcomes are heart rate variability (HRV) and BP. The study will also characterize physical activity and sleep to further contextualize stress experiences and response. This study was approved by the Virginia Commonwealth University Institutional Review Board.

### Participant eligibility

Eligible participants will meet the following conditions: 1) be adults aged 18–39 years old; 2) identify as Black or African American; 3) identify as a woman; and 4) have a measured blood pressure of <140/90 mmHg at baseline. Including women with normal BP (<120/80 mmHg) and elevated BP/Stage I hypertension (≥120/80 mmHg to 139/89 mmHg) will inform potential variation in treatment response and direct design efforts towards primary or secondary intervention. Additional details sare listed in **Table 1**.

**Table 1:** Study Inclusion and Exclusion Criteria.

*Inclusion Criteria*
• Self-identify as a woman • Self-identify as African American (AA) or Black • Measured blood pressure (BP) of <140/90 mmHg. • Age 18 to 39 years old
*Exclusion Criteria*
Laboratory & medication exclusions • Are currently taking medication for hypertension Medical history exclusions • Cognitive impairment • Hospitalization for any psychiatric disorder within the past 12 months • History of schizophrenia, a psychotic disorder, or any organic brain syndromes • Bipolar disorder that is currently unmanaged • Current substance use (AUDIT >16) • Pre-existing heart disease or heart condition Pragmatic or potentially confounding exclusions • Unwilling/unable to use a personal device with EMA application or wear an ECG patch (including severe allergies to the patch’s adhesive) • Currently participating in a potentially confounding research study (e.g., stress reduction, BP management)

### Participant Recruitment

Beginning on February 13, 2024, recruitment and data collection were scheduled for a 24-month period using various community-based recruitment strategies. Recruitment is expected to conclude by August 8, 2025; data collection will conclude by September 30, 2025. Preliminary results are expected by March 2026. Digital advertisements will be distributed through university-wide listserv communications and email blasts, social media channels, word-of-mouth, local businesses and virtual/paper flyers. Flyers will be distributed in physical locations around Richmond, including medical centers, academic offices, community health hubs, and local businesses.

### Study data collection and contact schedule

Eligibility, baseline, and follow-up data are collected in-person and via online HIPAA compliant software and applications, and wearable devices (**Table 2**).

**Table 2:** Study Measurement Components and Schedule.

Visit	Initial Screening	Final Screening	Orientation	Training Period	Baseline	Follow-Up	Closeout
Measurement Days	0	0	0	0	0	7	14
Type of visit	O	C	C	O	C	C	C
Screening questionnaire	X*						
Contact information	X						
Demographics form	X						
Informed consent		X					
14-day study period scheduled (baseline, follow-up and closeout)			X				
Dietary Screener			X				
MMSE			X				
Initial blood pressure measurements (3)			X				
Baseline survey				X*			
Medical history/baseline questionnaire					X		
PiLR EMA account activated via participant’s cellphone					X		
Actigraph watch activated					X		
Initial CardeaSolo patch placed					X		
Blood glucose (12-hour fasting)					X		
Height measurement					X		
Weight measurement					X		
Heart rate variability measurement					X		
5x daily EMA survey					X*	X*	X*
2x daily blood pressure monitoring					X*	X*	X*
Replacement CardeaSolo patch placed (initial patch removed)						X	
Exit survey							X

Note: O: online; C: clinic: *participant administered.

### Screening, enrollment, and orientation

**Fig 1:**
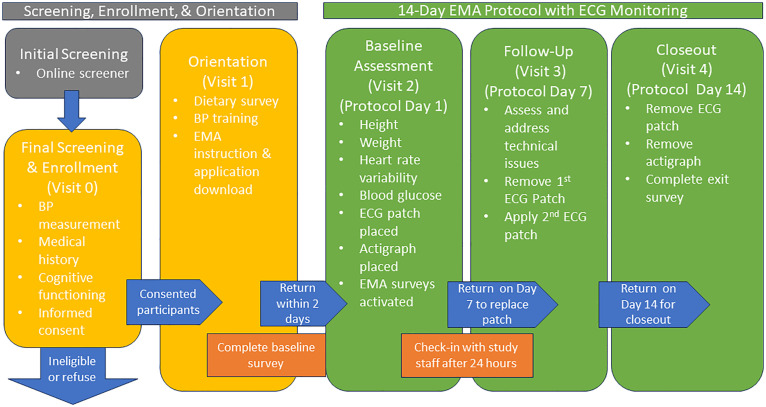
Overview of study procrdures.

The protocol consists of 4 in-person visits conducted at VCU (Fig 1). Initial screening involves a brief online survey via a HIPAA-compliant survey platform to verify initial eligibility. Upon confirmation of initial eligibility, trained study staff contact perspective participants to schedule an onsite screening and orientation visit to establish final eligibility. Written informed consent will be obtained from all participants before enrollment into the study.

Prospective participants are then presented with details of the study, including the study’s objectives and procedures, and the opportunity to ask questions of the study staff. For those who remain interested after the orientation presentation, final eligibility is established (i.e., blood pressure measured and medical history questions reviewed), additional study details provided, and informed consent obtained. Enrolled participants remain for additional orientation (Visit 1), including completing a dietary intake survey with the study staff, training and practice measuring their own blood pressure using a validated BP cuff provided by the study, and being introduced to and instructed to download the EMA application to their mobile phone.

After enrollment and before in-office Baseline Assessment (Visit 2), participants are invited via email to complete a self-administered baseline survey including questions on demographics, general health, including self-reported conditions [25], health behaviors [25], as well as validated measures of stress and stressors [24,26–30], coping strategies [31–34], and social drivers of health [35–38] via a HIPAA-compliant survey platform. The survey is designed to take approximately 30–35 minutes to complete (**Table 3**). Participants return to the lab within two days (48 hours) of the orientation visit to complete physiological baseline measures (i.e., height, weight, heart rate variability, and blood glucose), as well as report any complications in using the EMA application or BP cuff. Between Visits 1 and 2 participants undergo a “training period” where they engage the functionality of the application prior to engaging in their 14-day protocol. During this time, study staff are available to troubleshoot and address any technical issues. While it is preferable that participants return for Visit 2 within 2 days, participants may return up to 5 days after their orientation to begin their 14-day study period—a policy employed to accommodate the potentially fluctuating schedules of young adults.

**Table 3:** Baseline survey measurements of self-reported health and stressors.

Name	Description
Health conditions	Self-report if ever being told by a doctor or health professional that they have Diabetes/Prediabetes, Obesity, high blood cholesterol, depression, anxiety or uterine fibroids [25]
Health behaviors	Self-report: smoking, alcohol consumption, sleep, and physical activity [25]
Perceived Stress	Perceived Stress Scale (10-Item) (PSS-10) [24]
Depression	8-Item Depression Patient Health Questionnaire (PHQ-8) [26]
Anxiety	7-Item Generalized Anxiety Disorder Scale (GAD-7) [27]
Discrimination	Everyday Discrimination Scale [28]
Gendered Racial Microaggressions	Gendered Racial Microaggressions Scale [29]
Adverse Childhood Experiences	Adverse Childhood Experiences (ACE) Scale [30]
Resilience	6-Item Brief Resilience Scale [31]
Self-efficacy	New General Self-Efficacy Scale [32]
Emotional Support	4-Item PROMIS Emotional Support subscale [33]
Religiosity	2-Item Daily Spiritual Experience Scale [34]
Financial Strain	2 Financial strain items regarding current financial situation and ability to pay for basics [35]
Transportation	Transportation barriers item [36]
Food Insecurity	6-item United States Department of Agriculture Food Insecurity Survey [37,38]

### 14-Day protocol period

During Visit 2 (Protocol Day 1), participants will have their blood glucose, HRV, and height/weight baseline measurements assessed as well as have the ECG patch placed and receive a programmed actigraph watch. EMA surveys are activated during the visit. Study staff check in with participants within 24 hours of enrollment in the 14-day study period to ensure they have received the first batch of surveys via the application installed on their mobile phones and are completing the study activities per protocol. On Protocol Day 7 of 14, participants return to the lab for Visit 3 to have the first ECG patch collected and replaced with a new patch by the study staff and to discuss any concerns or questions. (ECG patches are designed to track 7 days of continuous data). At the conclusion of the 14-day data collection period (Visit 4, Protocol Day 14), participants return to the lab for a study debrief. The ECG patch and actigraph watch are collected. An exit survey is conducted regarding overall study experience, acceptability, and feasibility of the EMA procedure, specifically. Participants are compensated via cash or a check deposited directly into their bank accounts within 30 days of completing the study.

### Primary predictor

#### Stressors.

EMA will be used to measure *in vivo* stress experiences within naturalistic context. EMA data collection will be facilitated via the mobile application PiLR EMA (available through iPhone and Android) over a 14-day period using a combination of semi-random and event contingent sampling. Respondents will report the occurrence of various stressors: 1) semi-randomly (surveys randomly dispatched during pre-designated “waking hours” to avoid sleep disruptions) as prompted by the application; and 2) as self-reported events via surveys completed between prompts per participants’ experiences. Stress experience surveys capture a description of the stressor or stress event, who was affected (direct versus vicarious exposure), place of event, attitudinal and emotional reactions, and perceived social support. Participants are prompted to attribute the stress event to various types of factors (e.g., race, gender, family, some other factor). In addition, daily baseline and bedtime surveys assess sleep patterns, including quantity and quality of sleep, current emotional state, feelings of depression, feelings of anxiety, engagement in self-care or physical activity, coping, and experiences of a series of common daily hassles [24,25,39]. Validated measures of stress and other related measures [26,27,40]. (**Table 3**) will be ascertained at baseline via a HIPAA-compliant survey platform before the start of the 14-day study period to further contextualize the EMA survey data.

### Primary outcomes

#### Heart rate variability.

Heart rate variability is a useful measure of the complex and dynamic oscillations of a healthy heart, which is subject to sudden psychological and physiological changes. It represents a compilation of regulatory functions of the blood pressure, the autonomic nervous system, the heart, gut, and blood vessels [41]. When assessed alongside EMA, HRV can help assess antecedent cues for mental health symptoms [42]. Baseline heart rate variability will be recorded using the BIOPAC system during Visit 2. A three-lead ECG configuration will be applied, with an electrode placed on the medial side of each ankle, and to the right forearm as participants lie in a supine position remaining as still as possible.

During the 14-day study period, HRV will be assessed via CardeaSolo© patches—an ambulatory ECG monitoring system. The patch will be applied during Visit 2 and worn continuously for seven consecutive days. Once the skin has been cleaned and abraded, the patch is placed at approximately the third intercostal space on the left side of the chest. A return visit is necessary for application of a new sensor to be worn for a second round of seven days (Visit 3).

#### Blood pressure.

During the orientation meeting (Visit 1), three resting BP measurements will be taken by trained study staff, to confirm participation eligibility using an GE Carescape V100 monitor and Omron© Blood Pressure cuff (Series 3). These readings also serve as baseline assessment. Participants will be instructed to rest quietly for three consecutive minutes prior to the first reading. A Gulick tape measure will be used to assess arm circumference at the upper-arm to the nearest 0.1 cm to ensure proper cuff size. The three BP measures will be averaged to calculate mean systolic and diastolic blood pressure using an appropriately sized cuff.

Participants will be provided with a similar Omron© Blood Pressure Monitor (Series 3) to be used for at home blood pressure monitoring during the 14-day data collection period. Three home blood pressure measurements will be taken twice a day: at the conclusion of the morning survey, and at the conclusion of the end-of-day survey. Measurements will be reported by participants in the PiLR app.

### Other measurements

#### Life’s essential eight.

Additional variables related to the AHA’s “Life’s Essential Eight” for ideal cardiovascular health will be measured, including diet, physical activity, blood glucose, and weight, as will self-reported health behaviors and conditions, including current smoking and high cholesterol (**Table 3**) [43].

#### Diet.

Participants will complete a 16-item dietary intake survey facilitated by study staff during orientation (Visit 1), which includes consumption of olive oil, leafy greens, other fruits and vegetables, breads and pasta, beans, fast food, and alcohol [44].

#### Physical activity and sleep.

To assess physical activity and sleep, the ActiGraph GT9X Link will be used. The ActiGraph is a wearable device that contains an accelerometer and a light sensor, and is similar in size to a wristwatch. Participants will be provided with the ActiGraph device during Visit 2 and will wear it on their non-dominant wrist for the duration of the study protocol. In addition, physical activity is reported in the baseline survey, including number of days physically active per week and number of minutes physically active per day. Sleep duration is recorded by the Actigraph, assessed in the baseline survey via the item, “on average, how many hours of sleep do you get per night?”, and via the daily EMA survey, including previous night’s bedtime, wake time on the current day, and self-rated sleep quality. Periods of sleep will also be detectable via the continuous ECG reading.

#### Blood glucose.

A finger prick will be conducted to assess fasting blood glucose levels during Visit 2. Alcohol wipes will be used to thoroughly clean the participant’s fingers prior to the prick. A lancet will be used to puncture the skin of the finger, and trained study staff will take care to direct the stick to the top of the finger, rather than the side or the tip. Participants will be instructed to fast for 12 hours before Visit 2.

#### Height and weight.

During Visit 2, participants will have their height and weight measured by trained study staff using standardized height measurement and the Tanita BWB-800 scale, respectively. These values will be utilized to calculate body mass index (BMI) using the formula weight in kg/ height in m^2^.

#### Smoking.

Lifetime and current smoking will be assessed via baseline survey, including having smoked at least 100 cigarettes in life, last time smoked a cigarette, and living with anyone smoking indoors [25].

#### Blood cholesterol.

High cholesterol is self-reported via the baseline survey, “Have you ever been told by your doctor or health professional that you have high blood cholesterol?” [25].

### Adherence

Adherence to the protocol will be assessed based on the completion of EMA surveys via the PiLR application as well as days wearing the ECG patch and actigraph watch. Participants will receive up to 3 reminders in the EMA application to complete unanswered survey prompts as they are administered to promote completeness of data. Regular contact with study staff, including periodic phone calls and the mid-protocol check-in on Protocol Day 7, to address technical issues and other challenges will promote adherence.

### Safety

After enrollment in the study, participants are surveilled for adverse events through scheduled points of contact and follow-up (Fig 1) as well as ad hoc events are reported by participants. Adverse event forms will be completed that meet the protocol event criteria. Participants are monitored for extreme blood pressure values and severe allergies throughout the screening and follow-up visits.

### Analysis plan

Descriptive statistics will be used to examine the study sample by sociodemographic and baseline physiological characteristics (e.g., HRV and BP). We will use bivariate and multivariate analyses to examine the associations between stressors and physiological outcomes.

For bivariate analyses, we will assess stress exposure in terms of types (general, attributions to race, gender, or the intersection of the two), frequency, and attributions in the overall sample as well as by baseline HRV and BP using tests appropriate for the operationalization and distribution of measures (e.g., McNemar’s, paired t-test).

For multivariate analyses, within-person, the stress event will be used as a predictor of physiological stress response for the concurrent time of measurement and for some number of future measurements of stress response. The coefficients for these effects will provide information on the time it takes for return to baseline. To account for stressors associated with race or discrimination, the interaction between stressor and event type will be included. All effects will be modeled as random effects to allow for between-subject variables (e.g. race, gender, HRV) to moderate the effect of stressor events and event type. The model can be specified as a two-level model, with parallel equation for the remaining random effects. All analyses will be completed using the R statistical software [45] and the lme4 package [46] using an alpha of 0.05.

### Sample size and power

The projected sample size is 50 participants. Power analysis via Monte Carlo simulation with 500 replication per condition found this value to be sufficiently large to detect modest effects for both within- and between-subjects. Coefficient values were varied to estimate the empirical power for the proposed model. For within-subjects, we will have at least 0.80 power to detect a 0.10 standard deviation increase in physiological response. For between-subjects’ effects, we will have at least 0.80 power to detect a 0.20 standard deviation change in the random effects. These correspond to small effects for within- and between-subjects.

## Discussion

DYNAMIC assesses the in vivo stress experiences of young Black women in association with physiological outcomes, including heart rate variability and blood pressure. This study addresses current gaps in the literature regarding the effects of general and social stressors in real time and context towards the development of effective interventions for Black women—a high stress, high cardiovascular risk population. During a 14-day study period, this protocol concurrently measures stress experiences and physiological and physical outcomes by leveraging technology to discern stressors and coping stressors as they occur contextually. These data will be foundational for identifying which types of stressors may be correlated with chronic and prolonged stress response—a noted cardiovascular risk factor. Investigating these stressors during young adulthood offers both a critical period of notable and potentially stressful life transitions (e.g., family, education, career) as well as prime opportunity for the adoption of healthy lifestyle behaviors to promote primordial prevention and early intervention towards improving cardiovascular longevity within a group with notably earlier onset of cardiovascular risk factors, including high blood pressure [47].

This study also has some limitations. First, the primary outcome—HRV can be difficult to interpret; however, it is a very useful tool in predicting morbidities from stress, depression, anxiety, and inflammation [41,42]—conditions that promote autonomic imbalance and increase allostatic load—which is related to the core rationale of this study. Our interdisciplinary team includes experts in exercise physiology and cardiology with extensive experience in interpreting clinically significant changes in HRV. The ambulatory ECG monitors were selected over monitors of other cardiovascular functioning, such as BP, because of the ease of implementation for valid and continuous monitoring without interruption to activities of typical daily living [48]. Second, ambulatory blood pressure monitoring is the gold standard; however, we utilize participant self-measurement of blood pressure as a secondary outcome measure. We decided against using ambulatory blood pressure monitoring due to previously noted physical and social discomforts [49]—concerns potentially heightened within the context of young adulthood. Instead, participants were trained to use home BP cuffs, watched study staff model the behavior, and practiced with study staff to ensure confidence in their own skills. Self-measured blood pressure has to be shown to be a better predictor of cardiovascular morbidity and mortality over time than single-point office measurements [50,51]. In addition, the practice promotes active engagement in the process [52], which could promote better overall awareness of cardiovascular risk factors and health in this population. This skill (i.e., training in accurate blood pressure self-measurement) and tool (i.e., provision of a validated cuff) could promote continued self-monitoring, which could benefit participants’ cardiovascular health beyond the life of the study. Despite these limitations, this study is the first to employ EMA alongside physiological monitoring to study unique stress experiences and reactivity among young Black women through the lens of healthy cardiovascular aging.

DYNAMIC is an innovative observational study examining the association of *in vivo* stressors in the lived experiences of young adult Black women with cardiac response within natural environments, including neighborhood and social contexts. The data collected from this study will be used to inform tailored interventions that are responsive to the stress needs of young Black women with regards to heart health and healthy cardiovascular aging.
